# Effect of Short-Term Stress and Interaction of Salinity and Ammonia-N Levels, Associated With Food Deprivation on Fatty Acid Profile and Body Composition in Nile Tilapia (*Oreochromis niloticus*)

**DOI:** 10.1155/anu/8840365

**Published:** 2025-01-06

**Authors:** Eisa Ebrahimi, Javad Motamedi-Tehrani, Rahim Peyghan

**Affiliations:** ^1^Department of Natural Resources, Isfahan University of Technology, Isfahan 84156–8311, Iran; ^2^Faculty of Veterinary Medicine, Shahid Chamran University of Ahvaz, Ahvaz, Iran

**Keywords:** aquaculture industry and Iran, DHA, EPA, tilapia, water quality criteria

## Abstract

High levels of nitrogen compounds can lead to acute toxicity in aquatic organisms. Ammonia, a by-product of protein breakdown, is the most prevalent contaminant in freshwater environments. Increasing salinity in water sources can cause fluctuations in salinity levels within breeding ponds. The interaction of these elements can occur in breeding ponds, significantly impacting the physiology and quality of the aquatic products. The purpose of this study was to examine the relationship between salinity and ammonia-N stress and their effects on the quality and fatty acid profile of tilapia fish (*Oreochromis niloticus*). The fish were divided into 12 distinct treatment groups, each characterized by varying salinity levels (0, 4, 8, and 12 ppt) and different concentrations of ammonia-N (0, 50% of 50% lethal concentration [LC_50_]-96 h, and 30% of LC_50_-96 h) arranged in a factorial design. The calculated LC_50_-96 h for ammonia-N was 0.86 mg/L. Significant increases were observed in cortisol and glucose levels associated with various salinity treatments and ammonia levels. The levels of carcass protein in the salinity treatments (4, 8, and 12 ppt) did not show any significant differences when compared to the control treatment. However, the protein percentage at 50% of LC_50_-96 h of ammonia-N was lower than that of the control treatment. In salinity treatments and ammonia levels (50% and 30% of LC_50_-96 h of ammonia-N), a significant increase in the percentage of lipid, highly unsaturated fatty acids (HUFA), eicosapentaenoic acid (EPA), and docosahexaenoic acid (DHA) was observed. To draw the conclusion, our assessment indicates that a salinity concentration of 8 ppt over a 96-h period without feeding has produced positive effects on the quality of tilapia carcasses.

## 1. Introduction

Aquaculture serves as a method for producing safe and nutritious food for human populations [1]. Freshwater fish is a vital protein source, offering not only protein but also a variety of nutritious components, such as healthy fats [2]. Fish populations' health, survival, and success are greatly impacted by lipids [3, 4]. These molecules play a well-established role in fish growth, functioning as energetic, structural, hormonal, and biochemical precursors for eicosanoids [5]. Therefore, fish play an important role in the structure of the human food pyramid.

Maintaining the integrity and functionality of membranes is vital for proper growth and development, and polyunsaturated fatty acids (PUFAs) play a critical role in this process. [6, 7]. Many health benefits can arise from fatty acids, such as the prevention of coronary heart disease, cardiovascular disease, rheumatoid arthritis, depression, postpartum depression, cancers, diabetes, and anti-inflammatory action [4, 8]. Fish has been widely accepted as the most significant and readily accessible source of health-promoting omega-3 long-chain PUFAs (LC-PUFAs) intended for human utilization [9–11]. Nevertheless, as a traditional source of fat required for the production of farmed fish high in n-3 LC-PUFAs, the aquaculture feed industry is increasingly viewing fish oil as an unsustainable and impractical practice from an environmental and economic perspective [12, 13]. It has been reported that n-3 PUFAs, particularly docosahexaenoic acid (DHA) and eicosapentaenoic acid (EPA), are vital for supporting the growth of both the immune system and the nervous system of the brain, in addition to their role in reducing the risk of cardiovascular disease [14].

The regulation of fatty acids in fish is affected by environmental conditions, including salinity, temperature, and the changing seasons [15–19]. Fish regulates physiological changes resulting from changes in salinity by modifying the dynamic balance of osmotic pressure, which involves controlling the fluidity and permeability of cellular membranes through changes [20]. The synthesis of fatty acids, particularly LC-PUFAs, can be influenced by salinity, primarily through the enzymatic activity of desaturase and elongase proteins involved in fatty acid metabolism [21].

Among nitrogenous compounds, ammonia stands out as a significant pollutant in water bodies. It is one of the most toxic agents affecting aquatic organisms. In water, ammonia is present in both its unionized form (NH_3_) and its ionized form (NH_4_^+^), with the distribution of these forms influenced by pH, temperature, and salinity levels [22]. Exposure to significant amounts of ammonia results in a rapid increase in ammonia levels within the blood of fish [23]. As a result, this may lead to the accumulation of harmful concentrations of ammonia within the fish's body. Ammonia has a significant impact on the growth of aquatic organisms [24], reduces their resistance to diseases [25], causes physiological complications [26–28], induces oxidative stress [29–31], and can ultimately result in mortality in aquaculture environments [22]. Therefore, reducing the amount of this toxic substance in aquaculture is essential

Iran experiences annual rainfall that is less than one-third of the global average, primarily because of its positioning in a semiarid zone. Additionally, there is insufficient coordination between the timing and geographical distribution of rainfall [32]. It has been predicted that Iran will be among the 27 countries that will experience an increasing water shortage until 2025 [33]. The quality and quantity of aquifers are negatively impacted by the uncontrolled use of groundwater [32]. It is possible that the salinity of surface and groundwater resources has risen based on the description provided [34, 35] and we may need attention to fostering fish adapted to salinity. As a result, ammonia-N poisoning could potentially take place at diverse salinity levels in our ecosystem. Furthermore, it is significant to observe that the changes in the fatty acid composition of *Oreochromis niloticus* happen concurrently with variations in salinity and ammonia levels.

Nile tilapia, *O. niloticus*, is notably well-adapted to brackish water environments, where it demonstrates superior growth compared to freshwater conditions [36, 37]. Tilapia is known for its ability to transform dietary fatty acids into long-chain fatty acids [38, 39]. In dry and water-deficient areas such as Iran, Nile tilapia is an essential choice as a cultured freshwater fish because it is one of the most widely [40, 41] and successfully cultured freshwater fish.

The examination of fatty acids in aquaculture research is predominantly carried out in two main areas of study. The two fields of research focus on either the impact of dietary supplements on the fatty acid composition in fish [42–46] or the influence of incorporating fatty acids into the diet to enhance the resilience of fish to environmental stressors over an extended duration, such as 4 weeks [47–49]. Given the limited understanding of how stress influences carcass quality and fatty acid profiles in fish, this article explores the effects of environmental stress on these aspects. Therefore, the main issues addressed in our research were the interaction effect of ammonia and salinity stress on the body composition and fatty acid profile of Nile tilapia during a 96-h period with food deprivation.

## 2. Materials and Methods

### 2.1. Fish

The standards and animal care committee of Shahid Chamran University of Ahvaz guided the performance of this research. We developed and executed this research project in accordance with the framework proposed by Motamedi-Tehrani et al. [50]. A fish breeding center in Qom city (Qom, Iran) provided us with juveniles of the Nile tilapia (*O. niloticus*), and then oxygen-enriched cellophane bags were used to transport the fish to the Partako breeding center (Tiranchi village, Isfahan, Iran). Randomly selected fish (360 pieces), which measured 57.32 ± 16.7 g, 13.72 ± 2.6 cm, and appeared healthy were chosen for the experiments. In order to acclimate to the laboratory environment, the fish were placed in clean and thoroughly disinfected aquariums (100 L) for a duration of 7 days. The commercial food given to the fish consisted of 40% protein, 30% carbohydrates, 7% crude fat, 6% crude fiber, 10% ash, and 10% moisture twice a day. No mortality was observed during the adaptation process. This study was carried out in accordance with the guidelines established by the National Ethical Framework for Animal Research in Iran, receiving approval under the number EE/95.11.3.51112/scu.ac.ir.

### 2.2. Adjust Acidity

Based on the method of Motamedi-Tehrani and Peyghan [50], the tank water was treated with hydrochloric acid (0.4 N, Merck, Germany) and sodium hydroxide (0.4 N, Merck, Germany) in order to create buffer water with constant acidity [50]. After the addition of both acid and base, the tank water's acidity was analyzed. Over the course of 1 week, we conducted continuous monitoring of water acidity levels. Having been fixed the acidity of the water, this water was used for the entirety of the experiment [50].

### 2.3. Quality Criteria of Water

Using the Köldahl system and titration, the evaluation of total ammonium nitrogen (TAN) was carried out [51]. We assessed the concentration of NH_3_ by employing the standard table in conjunction with the relevant temperature and pH values [50], in accordance with the approach outlined by method of Boyd [52]. Electrical conductivity (EC) and acidity were measured using the EC meter (conductivity meter 4310, Jenway, UK) and the pH meter (744 pH meter, Metrohm, UK), respectively [51].

### 2.4. Determination of 50% Lethal Concentration (LC_50_) of Ammonia in Fish

An investigation was conducted to determine the LC_50_ using a static method by refreshing the water every 24 h and substituting the required levels of concentration. We implemented two separate concentrations of ammonium chloride (Merck, Germany) in our methodology, which resulted in a mortality rate of 100% and no losses [50]. Following this, six concentrations of ammonium chloride were determined exponentially within the ranges of the two preceding doses. Subsequently, following the period of adjustment, the fish were segregated into six distinct groups, each comprising 10 fish [53]. The fish underwent a 96-h examination period, during which the mortality rate was documented at 24-h intervals [54]. Throughout the duration of the experiment, both the temperature and pH were maintained at a constant level. The LC_50_ was determined at 24, 48, 72, and 96 h using probit software following the recording of mortality [22].

### 2.5. Experimental Design

In our study, we selected 12 treatments in total, which comprised three distinct concentrations of ammonia-N (0, 30%, and 50% of LC_50_-96 h) and four different salinity levels (0, 4, 8, and 12 ppt), along with their interactive combinations of ammonia toxicity and salinity, as detailed in Table 1 [50]. Half of the LC_50_-96 h was classified as acute poisoning, while 30% was categorized as subacute poisoning [22]. The treatment comprised of five repetitions for each session. The water was replaced daily and completely within a 96-h period. Daily measurements were taken for total ammonium-N, pH, and EC. Aeration was consistently conducted during the entire duration of the experiment [22].

### 2.6. Blood Sampling

Insulin syringes were utilized to obtain blood samples after the 96-h test period concluded. The 2-phenoxyethanol (0.3 mL L^−1^) was used to anesthetize the fish, which were then dried off with a towel [55]. Blood was obtained through the caudal vein using syringes that did not contain heparin. Serum was extracted by spinning down blood samples in nonheparinized tubes at a speed of 3000 × *g* for a duration of 10 min [50]. The liquid above the sediment was extracted, divided into smaller portions, and stored at a temperature of −70°C (Ultra Low Temperature Freezer, MDF-U71VC, Sanyo, Japan) until further testing procedures were conducted [55]. Following the sampling process, the intestines and internal organs of the fish were segregated.

#### 2.6.1. Glucose Assay

Owing to the production of the quinonemine which has a direct relationship with glucose levels, glucose level was measured using the autoanalyzer device (Autoanalyzer Biotechnica, BT 1500, Italy) and the Pars Azmoon laboratory kit (Glucose kit, Pars Azmoon, Iran).

#### 2.6.2. Cortisol Hormone Measurement

Cortisol hormone level was assayed using the Monobind test kit (Monobind Inc., USA) and ELISA method, based on the enzyme immunoassay including the primary reaction between antigen and antibody using a marker.

### 2.7. Poximate Body Composition

Upon completion of the experiment, all fish were weighed and subsequently euthanized. Five fish from each treatment group were then selected for further analysis. The whole body was used to measure factors. The proximate composition analysis involved the determination of moisture, crude protein, lipid, and ash content using the established standard procedures [56].

### 2.8. Determination of Fatty Acid Profile

The Unicam GC (Model 4600, Cambridge, UK) was utilized to ascertain the composition of fatty acids. The method developed by Bligh and Dyer [57] was employed for the identification of fatty acids via the oil extraction process. Fatty acid methyl ester was prepared, and then it was injected into the GC device [58].

### 2.9. Statistical Analysis

We implemented a factorial design (3 × 4) for this research, based on the methodological framework proposed by Motamedi-Tehrani [50]. Results were presented as means ± standard deviation (SD). All data were subjected to the Kolmogorov–Smirnov test to check their normality, followed by Levene's test for homogeneity of variance. All data were analyzed using two-way analysis of variance (ANOVA), save for physical and chemical factors of water assessed using one-way ANOVA. In order to evaluate differences among means, the data were analyzed by Tukey's post hoc test. The differences where *p*  < 0.05 were considered as statistically significant. Statistical analysis was performed by SPSS software version 24.

## 3. Results and Discussion

### 3.1. LC_50_ Values of Ammonia

The results obtained from measurement of LC_50_ ammonia-N were shown in Table 2. The LC_50_ ammonia-N of 24, 48, 72, and 6 h was 1.42, 1.365, 1.187, and 0.86 mg/L, respectively. Ammonia-N concentrations in our research were calculated based on the LC_50_-96 h.

### 3.2. Water Quality Factors

Based on Table 3, there were no significant differences in temperature and pH factors. In treatments 5, 6, and 7, NH_3_ concentration showed a significant difference compared to other treatments (*p*  < 0.05). NH_3_ concentration in acute ammonia poisoning-12 ppt of salinity, namely, treatment 8, was less than treatment 5 (*p*  < 0.05). NH_3_ measured in treatments 1, 2, 3, and 4 was the lowest value among experimental treatments.

The outcomes of the two-way ANOVA indicated that the impact of ammonia-N poisoning, salinity levels, and their combination on cortisol and body composition, including moisture, total protein, lipid, and ash, were found to be statistically significant (Table 4).

**Figure 1:**
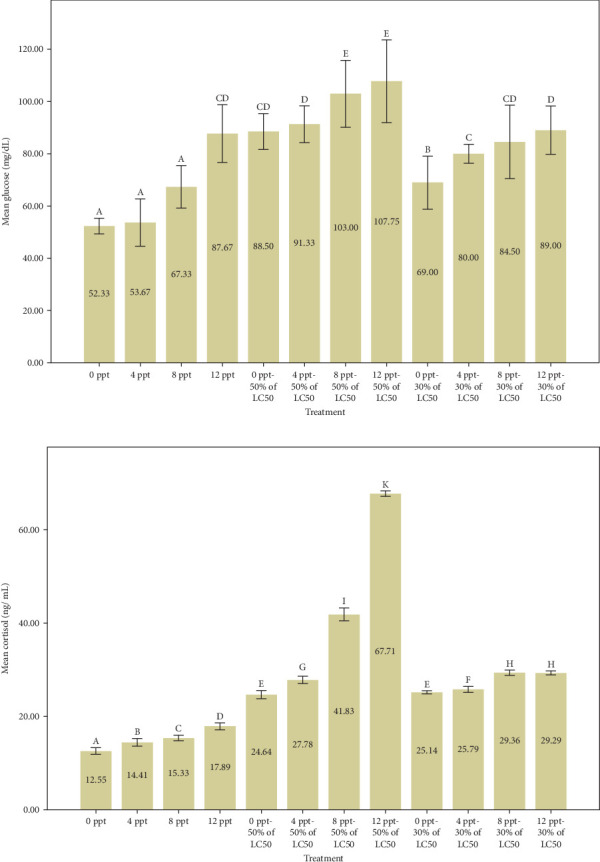
The effects of different levels of ammonia-N and salinity on serum glucose (a) and cortisol (b) levels of Nile tilapia after 96 h (mean ± standard deviation [SD], *n* = 5). The presence of different letters indicates a significant difference (*p*  < 0.05).

The findings from Figure 1 indicate a considerable rise in glucose and cortisol levels in cases of acute and subacute ammonia-N poisoning, specifically in treatments 5 and 9, in comparison to the control treatment. Additionally, it was observed that glucose and cortisol levels increased significantly with the rise in salinity among acute ammonia poisoning treatments (6, 7, and 8) in comparison to treatment 5 and among subacute poisoning treatments (10, 11, and 12) in comparison to treatment 9. The measurement of serum glucose, lactate, and cortisol concentrations is commonly regarded as a trustworthy method for evaluating stress resulting from different stress-related factors [59]. In general, the initial physiological response to stress involves the secretion of cortisol, while the subsequent responses include the release of glucose and lactate [60].

Similarly, Shi et al. [61] reported that the ammonia stress caused a significant increase in cortisol and glucose values in black sea bream, *Acanthopagrus schleglii*. Exposure to high levels of ammonia-N resulted in similar results in *Megalobrama amblycephala* [62], *Labeo rohita* [63], *Cyprinus carpio* [30], and *Anoplopoma fimbria* [64] that were consistent with our research results. It is obvious that the simultaneous increase of ammonia and salinity has a synergistic effect on the increased levels of stress indicators. Based on our results, it can be stated that the interaction effect of salinity and ammonia poisoning is synergistic to a great extent.

**Figure 2:**
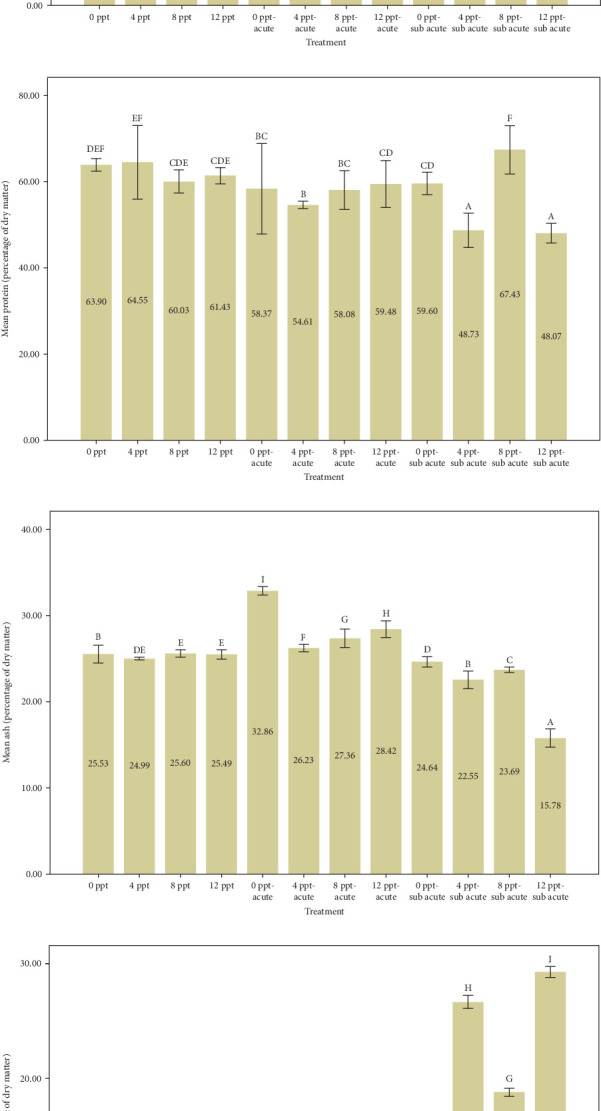
The effects of different levels of ammonia-N and salinity on body composition, moisture (a), protein (b), ash (c), and lipid (d), of Nile tilapia after 96 h (mean ± standard deviation [SD], *n* = 3). The presence of different letters indicates a significant difference (*p*  < 0.05).

Figure 2 provides a comparison of body composition in different treatments over 96 h. Based on the results of Figure 2, the findings indicated that the percentage of moisture in salinity treatments (i.e., treatments 2, 3, and 4 compared to the control) and interference treatments (i.e., treatments 6, 7, and 8 compared to treatment 5 and treatments 10, 11, and 12 compared to treatment 9) had a decreasing trend (*p*  < 0.05), while the moisture in acute and subacute ammonia poisoning treatments (treatments 5 and 9, respectively) was significantly higher than the control treatment. Considering the results of the protein measurement in Figure 2, a significant decrease was slightly seen between treatment 5 (acute ammonia poisoning) and control, but there were slight fluctuations between other treatments. There was a considerable increase in the percentage of ash in treatment 5 (viz., acute ammonia poisoning). Salinity treatments (i.e., treatments 2, 3, and 4) were not significantly different from the control treatment (Figure 2). Following the results of carcass ash percentage in Figure 2, increasing salinity in acute and subacute ammonia-N treatments (treatments 6, 7, and 8 compared to treatment 5 and treatments 10, 11, and 12 compared to treatment 9) showed a slight decrease in ash percentage. Figure 2 depicts the amount of lipid and that describes the lipid content was significantly increased in salinity (treatments 2, 3, and 4) and acute ammonia-N (i.e., treatment 5) compared to the control treatment.

In this study, we observed that the moisture content was significantly elevated in the ammonia treatments (5 and 9). However, the introduction of increased salinity levels (salinity treatments 2–4, along with interference treatments 5–8 and 9–12) resulted in a reduction of the carcass moisture content. This difference is probably due to the change in the osmotic pressure gradient in the environment. In hyperosmotic environments, freshwater fish tend to lose water from their bodies [65].

Increasing salinity has no significant effect on the amount of carcass protein. The results of protein measurement were different from the results of Wu et al. [66], in the article entitled “Culture salinity alters dietary protein requirement, whole body composition and nutrients metabolism related gene expression in juvenile Genetically Improved Farmed Tilapia (GIFT) (*Oreochromis niloticus*).” Chen et al. [67] reported the body protein percentage went up with increasing salinity, less than 10%, in cobia (*Rachycentron canadum*). In our opinion, a key factor contributing to the discrepancy between the outcomes of our article and the cited studies may be the length of the testing and feeding periods. Our test period was 4 days without feeding; nevertheless, the mentioned articles were done for 8 weeks with feeding. The amount of carcass protein in the 50% LC_50_-96 h of ammonia-N (treatment 5) had a significant decrease of about 9% compared to the control treatment. The reduction in carcass protein, coupled with a remarkable rise in cortisol and glucose levels in treatment 5 (Figure 1), when compared to the control group, suggests an inverse relationship between stress levels induced by ammonia treatment and protein content. This phenomenon is likely linked to the body's increased demand for amino acids to manage and cope with stress. In an article entitled “The effect of nitrite and nitrate treatment on growth performance, nutritional composition and flavor-associated metabolites of grass carp (*Ctenopharyngodon idella*),” Zhang et al. [68] mentioned nitrogenous waste can reduce the amount of protein, fat, and carbohydrates, which is consistent with the current results.

In the treatments involving varying levels of salinity and subacute ammonia-N, specifically treatments 9–12, a sinusoidal pattern was noted in the percentage of carcass protein, even though cortisol and glucose levels showed an increasing trend. The reasons behind these significant fluctuations remain ambiguous and warrant additional research; however, salinity is recognized as a fundamental factor in aquaculture [69], and it can influence homeostasis by creating changes in osmotic pressure levels [70]. Studies indicate that the isotonic point for juvenile tilapia is a salinity of 8 [66, 71, 72]. Therefore, it is likely that the increase in protein levels observed in treatment 11 results from these optimal isotonic conditions.

The percentage of carcass ash in intervention treatments expressed a significant decreasing trend with increasing salinity; however, one shows no significant change in salinity treatments 1–4 (Figure 2), along with the rising trend of cortisol and glucose (Figure 1). Lopresti [73] stated that stress can cause a decrease in minerals in the body (human or animal). In our research, it is believed that presumably the reduction of body ash may be triggered by certain levels of cortisol and glucose as stress.

In general, many factors such as the species, season, size, reproductive status, feed, density, and environmental conditions affect the amount of body fat [74]. As illustrated in Figure 2, generally speaking, increasing salinity levels alone and together with ammonia levels pronounced an upward trend in the lipid content. The percentage of lipid which jumped witnessed an upward trend from control to treatment 4. Our results were not consistent with ones of Woo and Kelly [75], in *Spams sarba*; Imsland et al. [76], in juvenile turbot (*Scophthalmus maximus*); Jarvis and Ballantyne [77], in shortnose sturgeon (*Acipenser brevirostrum*); and Chen et al. [67], in cobia (*R. canadum*), who reported that body fat decreases with increasing salinity. They believed that at salinities less than 10‰ (especially 8‰), less energy is probably stored as fat [78]. The reason for the difference between the data of the present research and those of the mentioned research probably is to a great extent related to the test period, adaptation to the conditions, and nutrition. It is essential to acknowledge that the increase in carcass fat was, to some degree, linked to heightened levels of cortisol and glucose [79–81]. This association was more pronounced in relation to the substantial fluctuations in cortisol and glucose that occurred during the intervention treatments.

The findings from the two-way ANOVA test, which examined the influence of salinity, ammonia, and their interaction on fatty acid factors, are detailed in Table 5. The analysis revealed that salinity, ammonia, and their interaction significantly impacted the fatty acid profile components.

Table 6 presents the percentage and classification of fatty acids derived from tilapia fish subjected to varying concentrations of ammonia and salinity, as well as their interactions. Due to the extensive range of treatments and the variety of fatty acids involved, a statistical comparison of the individual fatty acids was not conducted.

**Figure 3:**
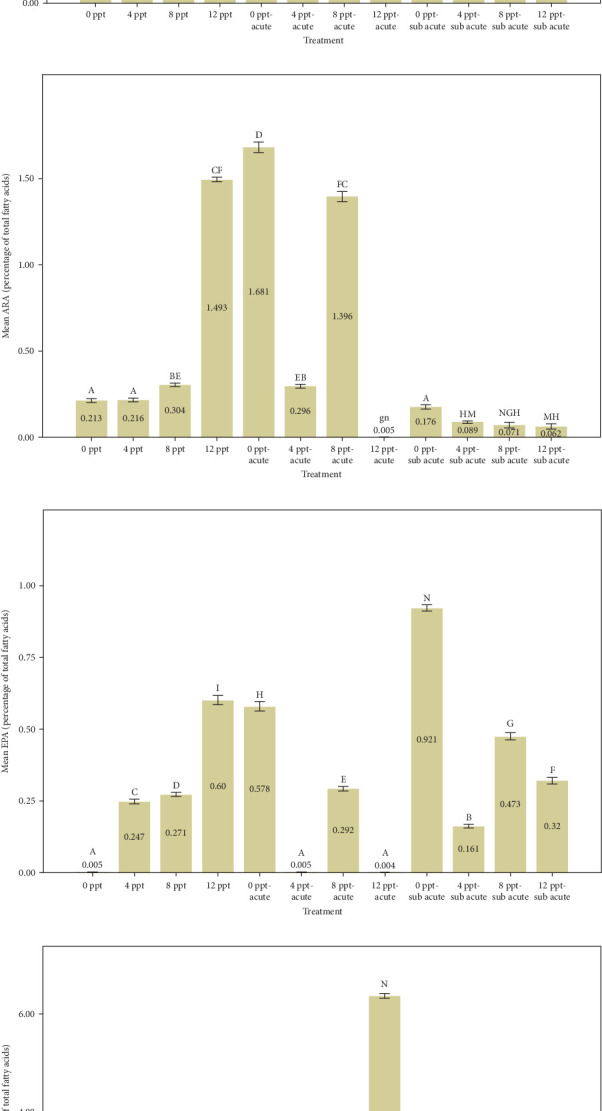
The effects of different levels of ammonia-N and salinity on factors of fatty acids of Nile tilapia, SFA (a), PUFA (b), HUFA (c), ARA (d), EPA (e), DHA (f), PUFA/SFA (h), and n-6/n-3 (g), after 96 h (mean ± standard deviation [SD], *n* = 3). The presence of different letters indicates a significant difference (*p*  < 0.05). ARA, arachidonic acid; DHA, docosahexaenoic acid; EPA, eicosapentaenoic acid; HUFA, highly unsaturated fatty acid; PUFA, polyunsaturated fatty acid; SFA, saturated fatty acid.

Based on the data presented in Figure 3, an increase in salinity from the control group to treatment 4 corresponds with a rise in PUFAs, in contrast to the behavior of saturated fatty acids (SFAs). This trend is statistically significant (*p*  < 0.05) and aligns with the observed elevations in cortisol and glucose levels depicted in Figure 1. The percentage of SFAs in acute and subacute ammonia treatments was considerably reduced compared to the control treatment; however, that of PUFAs shown in Figure 2 remained stable under 30%. Considering the higher increase of cortisol and glucose compared to saline treatments (Figure 1), in the intervention treatments (ammonia levels with increased salinity), both fatty acids had an almost increasing trend. The presence of SFAs is vital in decreasing the probability of heart attack occurrences [82]. Also, SFAs are involved in many processes such aslipogenesis, fat deposition, PUFA bioavailability, and apoptosis [82]. It seems that the salinity treatment of 8 ppt in interference treatments had better conditions to some extent compared to other interference treatments. Tilapia fish probably adapt better physiologically at 8 ppt [83].

Analyzing Figure 3 reveals that the percentages of highly unsaturated fatty acids (HUFAs), arachidonic acid (ARA), EPA, and DHA exhibited a remarkable increase in salinity treatments, specifically in treatments 2, 3, and 4, when compared to the control group. Conversely, the percentages observed in ammonia treatments, particularly in treatments 5 and 9, also demonstrated a gradual and significant rise relative to the control treatment. The changes of HUFA, EPA, and DHA in interventional treatments show no specific pattern, and the fluctuation levels of ones were more intense than other treatments (*p*  < 0.05).

The fatty acids play a crucial role in determining the quality and taste of meat [84]. Research has demonstrated that the composition of fatty acids in the body is significantly affected by dietary habits [85–87]. Tonial et al. [87] indicated that a feeding duration of 10 weeks may be adequate for altering the body's fatty acid profile; however, the experimental timeframe of the present study was limited to 96 h without any feeding. A progressive elevation in the levels of HUFAs, specifically EPA and DHA, was recorded, with significant increases noted in treatments 5 and 9, which pertain to acute and subacute ammonia treatments, respectively, in contrast to the control condition. N-3 PUFAs play a key role in anti-inflammatory activity in the body [88, 89]. Numerous studies have indicated that EPA and DHA play a crucial role in promoting anti-inflammatory responses and enhancing immune system function [90–95]. Based on Figure 1, cortisol and glucose levels were higher in ammonia treatments than the control treatment, and the amount of EPA, DHA, and HUFA was also higher in those treatments. Consequently, it may be essential to enhance immune and anti-inflammatory responses in ammonia treatments (specifically treatments 5 and 9) due to elevated stress levels. During the interference treatments (treatments 5–8 and 9–12), the percentages of HUFA, DHA, and EPA exhibited irregular and sinusoidal patterns, even in the presence of high cortisol and glucose levels. The reasons behind these fluctuations are not fully understood and require additional research. Nevertheless, it is evident that a salinity of 8 ppt resulted in improved conditions during the interventional treatments related to salinity and ammonia. This observation can presumably be linked to the adaptability and physiological traits of tilapia in environments with a salinity of 8 ppt. Figure 3 illustrates the ratio of PUFA to SFA. There was a significant upward trend in salinity (2, 3, and 4) along with ammonia treatments (5 and 9) compared to the control treatment and interventional treatments (treatments 6, 7, and 8 compared to 5 and treatments 10, 11, and 12 compared to 9). The PUFA-to-SFA ratio is a vital nutritional element. Evidence suggests that increasing this ratio may contribute to a decreased risk of contracting COVID-19 [96]. Research has shown that a ratio of 1–1.5 can reduce the risk of COVID-19 [96], which is interesting in this research; despite short-term stress, the P:S ratio in salinity and even ammonia treatments is more than 1 (Figure 3).

The balance of n-6/n-3 PUFAs has been proven for normal physiological activities [97]. Figure 3 depicts n-6/n-3 PUFAs, which were significantly reduced in ammonia treatments (5 and 9) and salinity treatments (4, 8, and 12 ppt) except 8 ppt compared to the control treatment. It is believed that the optimal n-6/n-3 PUFAs for normal physiological activities are ~1–2:1 [98, 99]. Fortunately, the short-term exposure to salinity treatments does not appear to negatively impact the n-6/n-3 ratio.

## 4. Conclusion

The effect of short-term stress and the interaction of salinity and ammonia-N, on body composition and fatty acid profile, were investigated in Nile tilapia (*O. niloticus*) by the present study. Taking everything into consideration, we believe that the stress in the treatment of 8 g per liter was able to have favorable effects on the carcass quality, increasing the level of PUFA, HUFA, EPA, DHA, and ARA, during 96 h without feeding. It is recommended to explore the combined impact of salinity and ammonia stress on postmortem alterations, histopathological sections, and organoleptic factors in fish in shorter periods of time such as a few hours.

## Figures and Tables

**Table 1:** Experimental treatments during 96 h.

Treatment	Explanation
T_1_	Adjusted water in terms of acidity (control)

T_2_	4 ppt of salinity

T_3_	8 ppt of salinity

T_4_	12 ppt of salinity

T_5_	50% of LC_50_-96 h (as acute ammonia poisoning), 0 ppt of salinity

T_6_	50% of LC_50_-96 h (as acute ammonia poisoning), 4 ppt of salinity

T_7_	50% of LC_50_-96 h (as acute ammonia poisoning), 8 ppt of salinity

T_8_	50% of LC_50_-96 h (as acute ammonia poisoning), 12 ppt of salinity

T_9_	30% of LC_50_-96 h (as sub-acute ammonia poisoning), 0 ppt of salinity

T_10_	30% of LC_50_-96 h (as sub-acute ammonia poisoning), 4 ppt of salinity

T_11_	30% of LC_50_-96 h (as sub-acute ammonia poisoning), 8 ppt of salinity

T_12_	30% of LC_50_-96 h (as sub-acute ammonia poisoning), 12 ppt of salinity

**Table 2:** LC_50_ values of ammonia in different times in Nile tilapia.

Duration (h)	LC_50_ (mg/L)	95% confidence interval
24	1.42	(0.611–1.77)
48	1.365	(1.131–1.625)
72	1.187	(0.982–1.397)
96	0.86	(0.708–1.09)

**Table 3:** Water's physical and chemical characteristics over a period of 96 h (mean ± standard deviation, *n* = 4).

Treatment	Temperature (°C)	pH	NH_3_ (mg/L)	EC (ms)
1	27.8 ± 0.26	7.02 ± 0.02	0.047 ± 0.036^a^	0.52 ± 0.02^a^
2	27 ± 0.2	7.04 ± 0.06	0.04 ± 0.02^a^	7.5 ± 0.24^a,b^
3	28 ± 0.15	7.07 ± 0.03	0.02 ± 0.001^a^	14.79 ± 0.58^d,e,f^
4	27.8 ± 0.26	7.01 ± 0.02	0.09 ± 0.03^a^	19.2 ± 0.18^f^
5	27.76 ± 0.2	7 ± 0.01	1.62 ± 0.83^e^	3.6 ± 0.01^a,b,c^
6	28.13 ± 0.32	7.02 ± 0.04	1.62 ± 0.51^d,e^	9.6 ± 3.3^c,d,e^
7	28.03 ± 0.05	7.04 ± 0.4	1.11 ± 0.12^d,e^	15.41 ± 7.6^e,f^
8	28 ± 0.9	6.98 ± 0.02	0.73 ± 0.037 ^c,d^	17.12 ± 8^f^
9	27.86 ± 0.15	7.04 ± 0.05	0.45 ± 0.14^b,c^	1.44 ± 0.01^a,b^
10	27.92 ± 0.13	7 ± 0.07	0.59 ± 0.05^a,b^	8.44 ± 0.09^c,d^
11	28 ± 0.1	7.03 ± 0.03	0.54 ± 0.11^b,c^	15.96 ± 1.08^e,f^
12	27.9 ± 0.17	7.07 ± 0.035	0.71 ± 0.11^a,b^	19.87 ± 0.24^f^
*p* value	0.442	0.183	0.00	0.00

*Note:* The presence of different letters in each column indicates a significant difference (*p*  < 0.05).

**Table 4:** The *p* value derived from a two-way analysis of variance demonstrates the impact of ammonia-N level, salinity, and their combined effects on body composition and serum cortisol in Nile tilapia.

Variable	Moisture	Protein	Lipid	Ash	Cortisol	Glucose
Ammonia	0.00	0.00	0.00	0.00	0.00	0.00
Salinity	0.00	0.03	0.00	0.00	0.00	0.00
Ammonia × salinity	0.00	0.00	0.00	0.00	0.00	0.005

*Note:* A *p* value of less than 0.05 suggests a noteworthy contrast in the variable of interest.

**Table 5:** The *p* value derived from the two-way analysis of variance indicates the influence of ammonia-N concentration, salinity, and their interactions on the fatty acid composition in Nile tilapia.

Variable	SFA	PUFA	HUFA	ARA	EPA	DHA	PUFA/SFA	n-6/n-3
Ammonia	0.00	0.00	0.00	0.00	0.00	0.00	0.00	0.00
Salinity	0.00	0.00	0.00	0.00	0.00	0.00	0.00	0.00
Ammonia × salinity	0.00	0.00	0.00	0.00	0.00	0.00	0.00	0.00

*Note:p*  < 0.05 indicates a significant difference in the desired variable.

Abbreviations: ARA, arachidonic acid; DHA, docosapentaenoic acid; EPA, eicosapentaenoic acid; HUFA, highly unsaturated fatty acids; PUFA, polyunsaturated fatty acid; SFA, saturated fatty acid.

**Table 6:** Fatty acids in tilapia fish fillet that were measured and identified (percentage of the total fatty acids identified).

Variable	T_1_	T_2_	T_3_	T_4_	T_5_	T_6_
Fatty acid						
C14:0	2.03 ± 0.03	1.12 ± 0.006	1.19 ± 0.01	ND	0.96 ± 0.05	0.96 ± 0.02
C15:0	0.35 ± 0.009	0.32 ± 0.006	0.35 ± 0.009	0.32 ± 0.007	0.28 ± 0.01	0.23 ± 0.009
C16:0	22.15 ± 0.03	20.84 ± 0.11	23.1 ± 0.03	17.6 ± 0.42	21.65 ± 0.28	19.7 ± 0.16
C16:1	2.15 ± 0.4	2.71 ± 0.01	2.21 ± 0.01	2.74 ± 0.05	ND	2.54 ± 0.05
C17:0	0.25 ± 0.01	0.53 ± 0.03	0.61 ± 0.01	0.41 ± 0.01	0.56 ± 0.006	0.68 ± 0.003
C17:1	0.085 ± 0.01	0.15 ± 0.005	0.14 ± 0.006	0.16 ± 0.007	0.16 ± 0.006	0.18 ± 0.007
C18:0	9.45 ± 0.03	7.25 ± 0.02	7.88 ± 0.009	1.48 ± 0.01	5.34 ± 0.01	7.54 ± 0.02
C18:1(n-9)	35.37 ± 0.01	33.28 ± 0.01	30.13 ± 0.008	38.02 ± 002	39.11 ± 0.03	35.72 ± 0.02
C18:2(n-6)	21.18 ± 0.01	26.45 ± 0.05	26.81 ± 0.01	29.62 ± 0.06	22.69 ± 0.01	27.62 ± 0.03
C18:3(n-6)	0.46 ± 0.02	0.52 ± 0.006	0.53 ± 0006	0.76 ± 0.004	0.54 ± 0.005	0.59 ± 0.007
C18:3(n-3)	1.34 ± 0.002	1.39 ± 0.002	1.09 ± 0.003	1.57 ± 0.009	1.71 ± 0.006	1.54 ± 0.01
C20:0	ND	ND	ND	0.1 ± 0.003	ND	ND
C20:1	ND	1.28 ± 0.009	1.28 ± 0.05	1.69 ± 0.007	1.67 ± 0.02	1.14 ± 0.005
C20:3(n-3)	1.19 ± 0.006	0.38 ± 0.006	0.37 ± 0.009	0.58 ± 0.008	0.38 ± 0.009	0.21 ± 0.01
C20:3(n-6)	0.8 ± 0.007	1.29 ± 0.01	1.44 ± 0.05	ND	ND	0.91 ± 0.02
C20:4(n-6)	0.21 ± 0.004	0.21 ± 0.005	0.3 ± 0.004	1.49 ± 0.006	1.68 ± 0.01	0.29 ± 0.005
C22:0	0.38 ± 0.004	ND	ND	0.2 ± 0.003	0.58 ± 0.005	ND
C20:5(n-3)	ND	ND	ND	0.17 ± 0.006	ND	ND
C22:4(n-6)	ND	0.17 ± 0.002	0.31±.005	0.53 ± 0.003	0.49 ± 0.002	ND
C22:5(n-3)	ND	0.24 ± 0.004	0.27 ± 0.003	0.6 ± 0.008	0.57 ± 0.008	ND
C22:6(n-3)	0.6 ± 0.009	1.86 ± 0.029	2.08 ± 0.06	1.85 ± 0.03	1.36 ± 0.05	0.28 ± 0.01

	**T_7_**	**T_8_**	**T_9_**	**T_10_**	**T_11_**	**T_12_**

Fatty acid						
C14:0	0.95 ± 0.03	0.05 ± 0.007	1.53 ± 0.03	1.84 ± 0.02	2.35 ± 0.007	1.51 ± 0.01
C15:0	0.25 ± 0.007	ND	0.26 ± 0.008	0.11 ± 0.005	0.01 ± 0.001	0.25 ± 0.005
C16:0	21.32 ± 0.19	24.28 ± 0.24	20.17 ± 0.15	24.7 ± 0.25	24.58 ± 0.22	24.36 ± 0.16
C16:1	0.01 ± 0.009	ND	7.24 ± 0.07	6.39 ± 0.2	6.5 ± 0.33	5.5 ± 0.12
C17:0	0.45 ± 0.006	ND	ND	0.11 ± 0.005	0.31 ± 0.008	ND
C17:1	0.13 ± 0.01	ND	ND	0.06 ± 0.005	0.08 ± 0.007	ND
C18:0	5.55 ± 0.04	15.91 ± 0.05	2.35 ± 0.04	6.39 ± 0.009	5.88 ± 0.09	4.49 ± 0.007
C18:1(n-9)	33.07 ± 0.03	32.08 ± 0.001	39.05 ± 0.03	42.69 ± 0.04	40.45 ± 0.03	42.88 ± 0.04
C18:2(n-6)	31.27 ± 0.02	16.04 ± 0.01	20.12 ± 0.008	13.09 ± 0.005	12.09 ± 0.006	16.3 ± 0.01
C18:3(n-6)	0.42 ± 0.009	ND	0.7 ± 0.007	0.13 ± 0.006	0.38 ± 0.006	0.25 ± 0.03
C18:3(n-3)	1.03 ± 0.004	0.3 ± 0.004	0.47 ± 0.1	1.78 ± 0.01	1.76 ± 0.03	1.68 ± 0.01
C20:0	ND	ND	0.07 ± 0001	0.12 ± 0.003	0.2 ± 0.004	0.1 ± 0.003
C20:1	1.27 ± 0.001	1.6 ± 0.006	1.28 ± 0.03	0.7 ± 0.01	0.71 ± 0.01	0.6 ± 0.009
C20:3(n-3)	0.36 ± 0.004	3.57 ± 0.03	0.65 ± 0.01	0.29 ± 0.003	0.43 ± 0.006	0.3 ± 0.001
C20:3(n-6)	ND	ND	1.89 ± 0.007	0.7 ± 0.004	1.05 ± 0.007	0.57 ± 0.02
C20:4(n-6)	1.390.01	ND	0.17 ± 0.005	0.09 ± 0.002	0.07 ± 0.008	0.06 ± 0.005
C22:0	ND	ND	0.27 ± 0.003	ND	ND	0.08 ± 0.003
C20:5(n-3)	ND	ND	0.4 ± 0.003	ND	0.16 ± 0.006	ND
C22:4(n-6)	0.37 ± 0.004	ND	0.49 ± 0.003	0.18 ± 0.007	0.46 ± 0.006	0.26 ± 0.006
C22:5(n-3)	0.29 ± 0.003	ND	0.92 ± 0.005	0.16 ± 0.003	0.47 ± 0.005	0.32 ± 0.2
C22:6(n-3)	1.98 ± 0.01	6.36 ± 0.02	2.17 ± 0.009	0.58 ± 0.005	1.74 ± 0.03	0.91 ± 0.002

Abbreviation: ND, not detected.

## Data Availability

The data that support the findings of this study are available from the corresponding author upon reasonable request.
